# Embarrassment as a public vs. private emotion and symbolic coping behaviour

**DOI:** 10.3389/fpsyg.2024.1437298

**Published:** 2024-09-04

**Authors:** Meikel Soliman

**Affiliations:** Faculty of Management and Technology, Institute of Management & Organization, Leuphana University Lüneburg, Lüneburg, Germany

**Keywords:** emotion, public embarrassment, private embarrassment, symbolic coping, context

## Abstract

In dealing with embarrassment, individuals engage in symbolic coping behaviours (e.g., hiding one’s face by wearing sunglasses). Research investigated these behaviours when embarrassment is experienced as a public emotion (e.g., others present). Contrary, there is emerging evidence showing that embarrassment can be experienced as a private emotion (e.g., no others present) as well. This is why the present research seeks to enhance previous work on symbolic coping behaviours and investigates to what extent symbolic coping behaviours differ when embarrassment is experienced in public and private. First, the present study finds that individuals experience embarrassment as a private as well as a public emotion. Second, both types of embarrassment relate to symbolic coping behaviours. Third, while both types of embarrassment experience a preference for face-hiding products there are differences in symbolic coping behaviours. Fourth, the study transfers extant research to a different cultural context.

## Introduction

Research long assumed that individuals feel embarrassment when they show a behaviour that observers perceive to be inappropriate (Dahl et al., 2001). This is why, research has defined embarrassment as an emotional state “in which a person feels awkward or flustered in other people’s company or because of the attention of others, as, for example, when being observed engaging in actions that are subject to mild disapproval from others” (American Psychological Association; VandeBos, 2006). Typically, embarrassment has been thought of as a social emotion that arises in the presence of others, because individuals are concerned about their public image and being appraised by others (Edelmann, 1985; Dong et al., 2013). Contrary to most extant research, recent, albeit limited, studies contend that embarrassment is not necessarily an inherently public emotion. Instead, individuals can experience this emotion in a private context as well—without others present (Krishna et al., 2015). This is in line with research stating that embarrassment refers to actions that contradict individuals’ self-concept (Babcock, 1988).

Generally, individuals are highly motivated to avoid situations in which they might feel embarrassment and go to great length to cope with this negative emotion (e.g., Apsler, 1975). Individuals developed a lot of ways to deal with embarrassment (e.g., reframing the situations in one’s head, hiding, not buying an embarrassing product at all; Dahl et al., 2001; Grace, 2009; Krishna et al., 2015, 2019). These coping strategies are direct responses to embarrassment (Feinberg et al., 2012; Dong et al., 2013). This study diverges from these coping strategies and investigates symbolic coping behaviours (Dong et al., 2013).

Symbolic coping behaviour as a response to deal with embarrassment has not been intensively studied (see for an exception Dong et al., 2013). These type of behaviours refer to engaging in actions that metaphorically help in dealing with embarrassment. Embarrassment can elicit two different motives. First, individuals have a motivation to avoid embarrassing situations which metaphorically is expressed in the desire to “hide one’s face” (Dong et al., 2013, p. 2005). Second, feelings of embarrassment might result in a desire to show actions that restore the public image which is metaphorically expressed as “restoring one’s face” (Dong et al., 2013). In line, experiencing embarrassment in a public context results in individuals having a greater preference for face-hiding products (e.g., sunglasses) and face-restoring products (e.g., facial moisturizer; Dong et al., 2013). As research investigated symbolic coping behaviours when individuals experience embarrassment as a public and not as a private emotion, the present study poses the following research question: To what extent do symbolic coping behaviours differ when embarrassment is experienced as a public compared to private emotion?

In answering this question, the present study builds on the second study of Dong et al.’s (2013) seminal work on symbolic coping behaviours and embarrassment. The present research diverges from Dong et al. (2013) in the following ways. First, this study seeks to replicate findings by Dong et al. (2013), finding that public embarrassment relates to symbolic coping behaviours. Second, while Dong et al. (2013) used a Hong Kong Chinese sample, here a European sample is used, trying to replicate findings to a sample with a different cultural background. Third, whereas Dong et al. (2013, p. 2005) assumed that “*embarrassment is inherently a public emotion*,” this study diverges from this and assumes that embarrassment can also be experienced as a private emotion which is in line with emerging research (Krishna et al., 2015). Fourth, focused on face hiding and face restoring products as they relate to individuals’ tendency of face hiding and face restoring respectively, here also face-washing products are included, assuming that individuals have a tendency to “wash away” their felt embarrassment. Fifth, Dong et al. (2013) focused on coping behaviours and did not include if individuals have the tendency to actually “hide one’s face” (Krishna et al., 2019). In the present research, short scales to measure individuals’ tendencies to either “hide face,” “restore face” or “wash face” were developed which was suggested by previous research (Krishna et al., 2019). Against this background, this paper hopes to enhance the understanding of embarrassment as a public and private emotion and how symbolic coping behaviours relate to both types of embarrassment.

## Conceptual background

### Embarrassment as a public and private emotion

Daily, individuals use the terms embarrassment, guilt, and shame interchangeably (Giorgetta et al., 2023). Research, however, showed that while these emotions have some overlap they are also distinct emotions (see for a discussion Tangney et al., 1996) which is also shown in distinct neurological correlates (Bastin et al., 2016).

Predominant research defines embarrassment as a “social emotion whereby one feels an aversive state of abashment and chagrin associated with unwanted mishaps or social predicaments” (Krishna et al., 2019, p. 1). Classically, research suggests that to experience embarrassment individuals need an audience and that this emotion depends on being appraised by others (VandeBos, 2006; Krishna et al., 2015). This traditional view of embarrassment is what Krishna et al. (2015) term public embarrassment. This way of looking at embarrassment does not paint a full picture of this emotion.

There is emerging evidence, that to experience embarrassment individuals do not need an audience. In line, Babcock (1988, p. 460) defines embarrassment as an “unpleasant response to the recognition that one has acted in a way that is inconsistent with one’s persona, i.e., that one has violated one’s personal standards … not a fear that he has failed or fumbled in the eyes of another. Thus, even though embarrassment may seem as if it requires an audience, it is essentially a private matter ….” This supports the notion, that individuals experience embarrassment not only as a public emotion, but also without the presence of others. Recent research supports this view. In three studies, Krishna et al. (2015) showed that individuals can experience embarrassment without others being present. The authors call this type of embarrassment private embarrassment. Findings suggest that individuals experience private embarrassment similarly to public embarrassment. Along with these findings, increasing evidence suggests that individuals can also feel vicarious embarrassment, meaning individuals feel this negative emotion when they witness someone being embarrassed, respectively, doing something that is considered embarrassing (Ziegler et al., 2022).

Why do individuals feel privately embarrassed? To address this question, Krishna et al. (2015) developed a typology which they base on two dimensions: social context and appraisal. Regarding the social context, individuals can experience embarrassment with others present public embarrassment and without others present private embarrassment. The second dimension addresses the mechanism: appraisal. Embarrassment can be the result of being appraised by others (real or imagined) or being appraised by the self. The reason why individuals feel privately embarrassed is based on whether they imagine other individuals’ judgement due to a norm violation or their own judgement because their mishap does not fit their self-concept.

### Symbolic coping behaviours

As embarrassment is a negative emotion, individuals either try to minimize feeling embarrassed or avoid it altogether. This is why they developed coping strategies which are efforts that individuals apply to deal with stress (Lazarus and Folkman, 1991), which can be subsumed in two types of strategies. Cognitive coping strategies refer to changing the way individuals think about the embarrassing incident, when they cannot do anything to avoid the embarrassing situation (e.g., engaging in thought exercises, denying their feelings; Grace, 2007, 2009; Krishna et al., 2019). Recently, there is evidence to suggest that one way to deal with embarrassment is to dehumanize others. For instance, when going to the doctor’s office, patients deprive the doctor of being fully human (i.e., less capable of emotional reactions) to deal with embarrassment (Sun et al., 2023).

In the present research behavioural coping strategies are the focus. These strategies can include actions such as purchasing counterbalancing products in addition to the embarrassing product (Blair and Roese, 2013), buying from a less attractive salesperson, stealing, avoid buying the product altogether (e.g., see Krishna et al., 2019 for a review), and preferring to interact with service robots (Holthöwer and Van Doorn, 2023).

Symbolic coping behaviours belong to behavioural coping strategies and are based on the idea that concepts can have a physical and a psychological dimension (Dong et al., 2013). In the context of embarrassment, individuals use expressions such as “restoring one’s face” which can refer to actions that help in “regaining one’s self-esteem” in the eyes of others but also using facial cosmetics (Dong et al., 2013, p. 2006). Individuals rely on those symbolic coping strategies. For instance, when lying via e-mail, individuals have a greater tendency to wash their hands and those who orally told a lie where more likely of wanting to wash their mouth (Lee and Schwarz, 2010).

**Figure 1:**
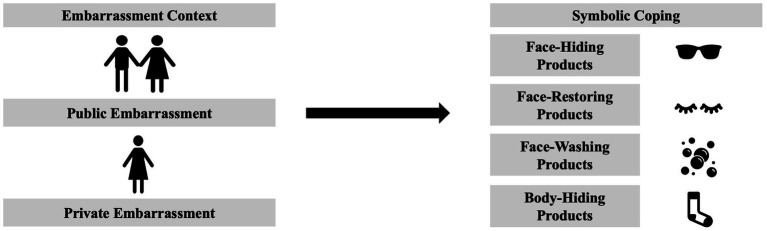
Experienced embarrassment and symbolic coping.

This is also the case for embarrassment (see Figure 1). After experiencing embarrassment, individuals want to avoid social attention and prefer wearing sunglasses to hide their face. They also prefer products that are symbolically related to restoring “their lost face” such as cosmetics (Dong et al., 2013). Due to limited research on symbolic coping behaviours (see Dong et al., 2013 for an exception) and private embarrassment (Krishna et al., 2015), research has not addressed how symbolic coping behaviours are related to private embarrassment.

Public and private embarrassment are different because the former requires an audience whereas the latter does not—likely resulting in different symbolic coping behaviours. When individuals are alone, there is no one to hide from making the motivation to publicly hide from others less applicable. Face hiding products might not alleviate the motivation to hide when experiencing private embarrassment. Instead, other products might be more useful, such as choosing washing products (Lee and Schwarz, 2010; Figure 1).

## Experiment: private vs. public embarrassment and symbolic coping

### Participants

Based on an *a priori* power analysis for an assumed conventionally ‘moderate’ population effect of *f = 0*.25, the standard 0.05 alpha error probability and a power of 0.80. The aim was to recruit at least 159 participants. Excess participants will be retained to achieve a higher power.

In total 189 participants were recruited. As preregistered (see OSF), participants who did not pass the implemented attention check (*n* = 15) and those who did not wrote an embarrassing story (*n* = 5) were excluded. This resulted in a total sample size of *N* = 169 (*M*_age_ = 24.65, *SD =* 9.69). In the final sample, 75.7% identified as female and 24.3% as male. For the assumed conventionally ‘moderate’ population effect of *f* = 0.25, the post-hoc power based on the final sample of *N =* 169 was 1−β = 83.01% which exceeds the threshold of having a power of 80% suggested by Giner-Sorolla et al. (2024).

### Study design and experimental manipulation

Depending on the condition, participants had to write about experiences in which they felt (a) publicly embarrassed (others present), (b) privately embarrassed (no others present), or (c) describe a typical Sunday (control condition). Participants were free to recall any embarrassing incident that they were able to come up with. In the embarrassing conditions, participants were asked to think about a situation in which they “*publicly (privately) felt a sense of embarrassment. In other words, is there an experience that embarrassed you (not) in the presence of other people?*” In the control condition, participants were prompted to “*Please think of a real experience that happened to you that describes a typical Sunday in your life. In other words, describe an everyday or typical Sunday for you*.” To help participants to describe the situation as in much detail as possible, they were provided with a few prompts such as “*What kind of physical reactions did you experience?*” and “*What did you think in this situation?*”

Similar to Dong et al. (2013) participants then participated in a seemingly unrelated study in which they were asked to imagine being in a department store having to indicate their willingness to visit the area in the department store where specific products are sold. Participants were presented with the following instruction “*Now let us move on to a completely different, everyday situation: Imagine that you are in a department store and would like to buy something.* Var*ious products are listed below. Please rate how likely it is that you will visit the part of the department store where each product is sold. Use the scale from* (1) *not at all likely to* (7) *very likely*.”

### Dependent measures

Participants were confronted with products for each product type relating to symbolic coping behaviours (see OSF for a full list of measures): (1) face-hiding products (e.g., sunglasses; 𝛼=0.73), (2) face-restoring products (e.g., facial moisturizer; 𝛼=0.90), (3) face-washing products (e.g., soap; 𝛼=0.85), and (4) body hiding products (e.g., gloves; 𝛼=0.68). Embarrassment was measured using an established three item scale (e.g., “*I felt embarrassed*.”; 𝛼=0.93; Dahl et al., 2001). Lastly, participants’ motivation (independently phrased) to hide the face (𝛼=0.59; “*I want to hide from people.*”), restore the face (𝛼=0.82; “*I want to feel better in my skin.*”), and wash the face (𝛼=0.69; “*I want to feel fresh and clean.*”) was captured by three items each.

### Engagement in writing task and experienced embarrassment

Engagement in the writing task did not differ between the public embarrassment (*M* = 5.72; *SD* = 0.98), private embarrassment (*M* = 5.43; *SD* = 1.23), and control group (*M* = 5.67; *SD* = 1.06), *F*(2,166) = 0.77, *p* = 0.466.

Experienced embarrassment differed, *F*(2,166) = 197.93, *p* < 0.001. The public embarrassment group felt more embarrassed (*M* = 5.51, *SD* = 1.35) compared to the private embarrassment group (*M* = 4.91, *SD* = 1.45), *t*(78.91) = 1.99, *p* = 0.050, and compared to the control condition (*M* = 1.57, *SD* = 0.96), *t*(82.30) = 18.10, *p* < 0.001. The private embarrassment group experienced more embarrassment compared to the control group, *t*(55.04) = 13.09, *p* < 0.001.

## Results

### Face-hiding products

**Figure 2:**
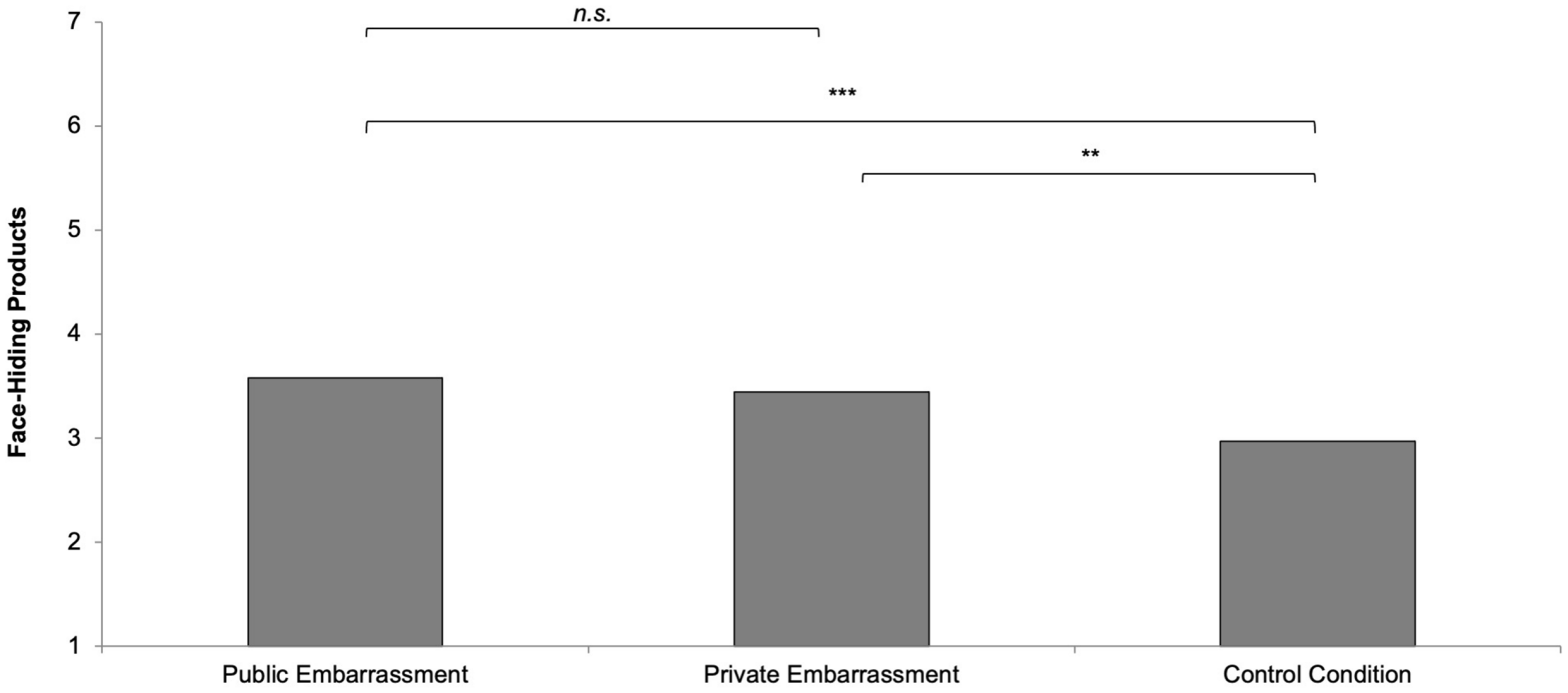
Face-hiding products as a function of social context.

The preference for face hiding products differed, *F*(2,166) = 6.96, *p* = 0.001, η^2^ = 0.08. The public embarrassment group had a similar preference for face-hiding products (*M* = 3.58, *SD* = 1.48) to the private embarrassment group (*M* = 3.44, *SD* = 1.25), *t*(166) = 0.52, *p* = 0.606, but more compared to the control condition (*M* = 2.97, *SD* = 1.16), *t*(166) = 3.44, *p* < 0.001. The private embarrassment group had a higher preference compared to the control group, *t*(166) = 2.59, *p* = 0.010 (see Figure 2).

### Face-restoring products

**Figure 3:**
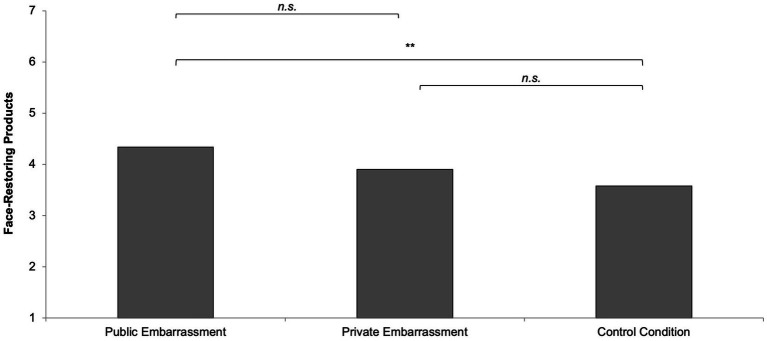
Face-restoring products as a function of social context.

The preference for face-restoring products marginally differed, *F*(2,166) = 2.46, *p* = 0.089, η^2^ = 0.03. The public embarrassment group had a similar preference for face-restoring products (*M* = 4.34, *SD* = 1.97) compared to the private embarrassment group (*M* = 3.90, *SD* = 2.10), *t*(166) = 1.08, *p* = 0.282, but more compared to the control group (*M* = 3.58, *SD* = 1.77), *t*(166) = 2.22, *p* = 0.028. The private embarrassment group did not differ from the control group, *t*(166) = 0.86, *p* = 0.391 (see Figure 3).

### Face-washing products

**Figure 4:**
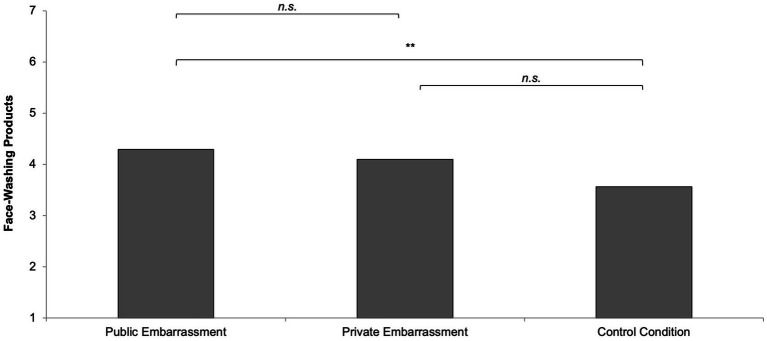
Face-washing products as a function of social context.

The preference for face-washing products marginally differed, *F*(2,166) = 3.01, *p* = 0.052, η^2^ = 0.04. The public embarrassment group had a similar preference for face-washing products (*M* = 4.29, *SD* = 1.91) compared to the private embarrassment group (*M* = 4.10, *SD* = 1.77), *t*(166) = 0.50, *p* = 0.615, but more compared to the control condition (*M* = 3.56, *SD* = 1.61), *t*(166) = 2.32, *p* = 0.022. The private embarrassment group did not differ from the control group, *t*(166) = 1.58, *p* = 0.116 (Figure 4).

### Body-hiding products

The preference for body-hiding products differed, *F*(2,166) = 7.20, *p* = 0.001, η^2^ = 0.08. The public embarrassment group had a greater preference for body-hiding products (*M* = 4.48, *SD* = 1.38) compared to the private embarrassment group (*M* = 3.83, *SD* = 1.36), *t*(166) = 2.36, *p* = 0.019, and compared to the control condition (*M* = 3.60, *SD* = 1.21), *t*(166) = 3.76, *p* < 0.001. The private embarrassment group did not differ from the control group, *t*(166) = 0.89, *p* = 0.377.

### Hiding, restoring, and washing tendencies

#### Face-hiding tendency

An ANOVA with the three groups (private embarrassment vs. public embarrassment vs. control group) as independent variable and face-hiding tendency as the dependent variable showed a significant effect, *F*(2,166) = 3.17, *p* = 0.044, η^2^ = 0.04. Contrast analyses revealed that the public embarrassment group had a similar face-hiding tendency (*M* = 2.54, *SD* = 1.12) to the private embarrassment group (*M* = 2.38, *SD* = 1.16), *t*(166) = 0.66, *p* = 0.509, but more compared to the control condition (*M* = 2.07, *SD* = 1.00), *t*(166) = 2.43, *p* = 0.016. The private embarrassment group did not significantly differ from the control condition, *t*(166) = 1.51, *p* = 0.134.

#### Face-restoring tendency

An ANOVA with the three groups (private embarrassment vs. public embarrassment vs. control group) as independent variable and face-restoring tendency as the dependent variable showed a significant effect, *F*(2,166) = 3.89, *p* = 0.022, η^2^ = 0.05. Contrast analyses revealed that the public embarrassment group had a similar face-restoring tendency (*M* = 5.06, *SD* = 1.38) compared to the private embarrassment group (*M* = 4.57, *SD* = 1.58), *t*(166) = 1.42, *p* = 0.157, and more compared to the control group (*M* = 4.25, *SD* = 1.75), *t*(166) = 2.79, *p* = 0.006. The private embarrassment group did not significantly differ from the control group, *t*(166) = 1.02, *p* = 0.311.

#### Face-washing tendency

An ANOVA with the three groups (private embarrassment vs. public embarrassment vs. control group) as independent variable and face-washing tendency as the dependent variable showed a significant effect, *F*(2,166) = 4.01, *p* = 0.020, η^2^ = 0.05. Contrast analyses revealed that the public embarrassment group had a similar face-restoring tendency (*M* = 5.29, *SD* = 1.18) compared to the private embarrassment group (*M* = 4.91, *SD* = 1.34), *t*(166) = 1.34, *p* = 0.183, and more compared to the control group (*M* = 4.62, *SD* = 1.37), *t*(166) = 2.83, *p* = 0.005. The private embarrassment group did not significantly differ from the control condition, *t*(166) = 1.15, *p* = 0.245.

## General discussion

The present research investigated in what way symbolic coping behaviours differ depending on embarrassment as public or private emotion. First, the findings corroborate extant research showing that individuals experience embarrassment as a private emotion as well as a public emotion (Krishna et al., 2015)—albeit to a lesser extent. Second, the present research can replicate findings from Dong et al. (2013) linking embarrassment to symbolic coping behaviours. Both the public and private embarrassment group experience a preference for face-hiding products. Third, while there seems to be an overlap of public and private embarrassment, there are also differences. While this corroborates findings from Dong et al. (2013) that individuals prefer face-restoring products after experiencing embarrassment as a public emotion, this does not seem to be the case for private embarrassment, illustrating that private embarrassment is different. It seems that symbolically losing one’s face pertains to an actual public setting. Fourth, this is also evident in the tendencies to hide, restore or wash faces which is only the case in the public embarrassment group. The private embarrassment group did not differ from the control group. This is highly interesting as it shows that these tendencies are inherent to embarrassment as public emotion and that embarrassment as a private emotion is different. Fifth, while Dong et al. (2013) conducted their study in a Hong Kong Chinese sample, the present study was conducted in Europe, showing that embarrassment as a public emotion is highly related to symbolic behaviours as well.

## Limitations and future research

The shortcomings of this research provide avenues for future research. First, as mentioned above, participants in this study were Europeans. While, the findings are similar to that of Dong et al. (2013), individuals need to know the metaphorical meaning of symbolic coping behaviours. To put it differently the “metaphorical meaning of these terms be known to individuals themselves.” (Dong et al., 2013, p. 2011). This means that the concept of hiding and losing one’s face, and washing away guilt or bad luck needs to be known by individuals. While these concepts might be universally known, culture differ in how pervasive these concepts are in society. This means that future research should conduct cross-cultural research, explicitly including a diverse sample. For instance, while the concept of losing face might be known in Europe, it might be more pervasive in Asian cultures (Dong et al., 2013). Second, the present research should be seen as a pilot study. This pilot study has a relatively small sample size. While this study exceeds the threshold of a power of 80% (Giner-Sorolla et al., 2024), future research might replicate this study using a larger and more diverse sample. Third, it might also be worthwhile to replicate the findings in the laboratory as well as in the field. It is interesting to investigate if the captured tendencies and product choices hold in the field as well. This way, research could tackle the question as to which extent symbolic coping is applied or if it is overridden by other coping behaviours. This is especially interesting as extant research shows that individuals have a variety of coping strategies when in the store such as buying additional products to cover up the embarrassing product (Blair and Roese, 2013).

## Data availability statement

The original contributions presented in the study are publicly available. This data can be found here: https://osf.io/6uem8/.

## Ethics statement

Ethical review and approval was not required for the study on human participants in accordance with the local legislation and institutional requirements. The participants provided their written informed consent to participate in this study.

## Author contributions

MS: Writing – review & editing, Writing – original draft, Visualization, Validation, Supervision, Software, Resources, Project administration, Methodology, Investigation, Funding acquisition, Formal analysis, Data curation, Conceptualization.

## References

[ref1] Apsler R. (1975). Effects of embarrassment on behavior toward others. J. Pers. Soc. Psychol. 32, 145–153. doi: 10.1037/h0076699

[ref2] Babcock M. K. (1988). Embarrassment: a window on the self. J. Theory Soc. Behav. 18, 459–483. doi: 10.1111/j.1468-5914.1988.tb00510.x

[ref3] Bastin C. Harrison B. J. Davey C. G. Moll J. Whittle S. (2016). Feelings of shame, embarrassment and guilt and their neural correlates: a systematic review. Neurosci. Biobehav. Rev. 71, 455–471. doi: 10.1016/j.neubiorev.2016.09.019 27687818

[ref4] Blair S. Roese N. J. (2013). Balancing the basket: the role of shopping basket composition in embarrassment. J. Consum. Res. 40, 676–691. doi: 10.1086/671761

[ref5] Dahl D. W. Manchanda R. V. Argo J. J. (2001). Embarrassment in consumer purchase: the role of social presence and purchase familiarity. J. Consum. Res. 28, 473–481. doi: 10.1086/323734

[ref6] Dong P. Huang X. I. Wyer R. S. Jr. (2013). The illusion of saving face: how individuals symbolically cope with embarrassment. Psychol. Sci. 24, 2005–2012. doi: 10.1177/0956797613482946 23938275

[ref7] Edelmann R. J. (1985). Individual differences in embarrassment: Selfconsciousness, self-monitoring and embarrassability. Personal. Individ. Differ. 6, 223–230. doi: 10.1016/0191-8869(85)90112-6

[ref8] Feinberg M. Willer R. Keltner D. (2012). Flustered and faithful: embarrassment as a signal of prosociality. J. Pers. Soc. Psychol. 102, 81–97. doi: 10.1037/a0025403, PMID: 21928915

[ref9] Giner-Sorolla R. Montoya A. K. Reifman A. Carpenter T. Lewis N. A. Jr. Aberson C. L.. (2024). Power to detect what? Considerations for planning and evaluating sample size. Personal. Soc. Psychol. Rev. 28, 276–301. doi: 10.1177/10888683241228328, PMID: 38345247 PMC11193916

[ref10] Giorgetta C. Strappini F. Capuozzo A. Evangelista E. Magno A. Castelfranchi C.. (2023). Guilt, shame, and embarrassment: similar or different emotions? A comparison between Italians and Americans. Front. Psychol. 14:1260396. doi: 10.3389/fpsyg.2023.1260396, PMID: 38192392 PMC10773588

[ref11] Grace D. (2007). How embarrassing! An exploratory study of critical incidents including affective reactions. J. Serv. Res. 9, 271–284. doi: 10.1177/109467050700900305

[ref12] Grace D. (2009). An examination of consumer embarrassment and repatronage intentions in the context of emotional service encounters. J. Retail. Consum. Serv. 16, 1–9. doi: 10.1016/j.jretconser.2008.02.004

[ref13] Holthöwer J. Van Doorn J. (2023). Robots do not judge: service robots can alleviate embarrassment in service encounters. J. Acad. Mark. Sci. 51, 767–784. doi: 10.1007/s11747-022-00862-x, PMID: 35463183 PMC9019535

[ref14] Krishna A. Herd K. B. Aydinoğlu N. Z. (2015). Wetting the bed at twenty-one: embarrassment as a private emotion. J. Consum. Psychol. 25, 473–486. doi: 10.1016/j.jcps.2015.02.005

[ref15] Krishna A. Herd K. B. Aydınoğlu N. Z. (2019). A review of consumer embarrassment as a public and private emotion. J. Consum. Psychol. 29, 492–516. doi: 10.1002/jcpy.1086

[ref16] Lazarus R. S. Folkman S. (1991). “The concept of coping” in Stress and coping: an anthology. eds. Monat A. Lazarus R. S. (New York, NY: Columbia University Press), 189–206.

[ref17] Lee S. W. S. Schwarz N. (2010). Dirty hands and dirty mouths: embodiment of the moral-purity metaphor is specific to the motor modality involved in moral transgression. Psychol. Sci. 21, 1423–1425. doi: 10.1177/0956797610382788, PMID: 20817782

[ref18] Sun Y. Wang X. Hoegg J. Dahl D. W. (2023). How consumers respond to embarrassing service encounters: a dehumanization perspective. J. Mark. Res. 60, 646–664. doi: 10.1177/00222437221130721

[ref19] Tangney J. P. Miller R. S. Flicker L. Barlow D. H. (1996). Are shame, guilt, and embarrassment distinct emotions? J. Pers. Soc. Psychol. 70, 1256–1269. doi: 10.1037/0022-3514.70.6.1256, PMID: 8667166

[ref20] VandeBos G. R. (2006). APA dictionary of psychology. Washington, DC: American Psychological Association.

[ref21] Ziegler A. H. Allen A. M. Peloza J. Norris J. I. (2022). The nature of vicarious embarrassment. J. Bus. Res. 153, 355–364. doi: 10.1016/j.jbusres.2022.08.038

