# Supplementary material for: Embarrassment as a public vs. private emotion and symbolic coping behaviour
Source: Front Psychol. 2024 Sep 4;15:1437298. doi: 10.3389/fpsyg.2024.1437298 (PMC11408275; doi:10.3389/fpsyg.2024.1437298)
Supplement: Supplementary file 1 [file Table_1.DOCX]

## Hiding, Restoring, and Washing Tendencies

**Face-Hiding Tendency**. Participants differed significantly regarding their face-hiding tendency, *F*(2,166)=3.17, *p*=.044. The public embarrassment group had a similar face-hiding tendency (*M*=2.54, *SD*=1.12) to the private embarrassment group (*M*=2.38, *SD*=1.16), *t*(166)=.66, *p*=.509, and more compared to the control condition (*M*=2.07, *SD*=1.00), *t*(166)=2.43, *p*=.016. The private embarrassment group did not significantly differ from the control condition, *t*(166)=1.51, *p*=.134.

**Face-restoring Tendency**. Participants differed significantly regarding their face-restoring tendency, *F*(2,166)=3.89, *p*=.022. Participants in the public embarrassment group had a similar face-restoring tendency (*M*=5.06, *SD*=1.38) compared to the private embarrassment group (*M*=4.57, *SD*=1.58), *t*(166)=1.42, *p*=.157, and more compared to the control condition (*M*=4.25, *SD*=1.75), *t*(166)=2.79, *p*=.006. The private embarrassment group did not significantly differ from the control condition, *t*(166)=1.02, *p*=.311.

**Face-Washing Tendency**. Participants differed significantly regarding their face-washing tendency, *F*(2,166)=4.01, *p*=.020. Participants in the public embarrassment group had a similar face-restoring tendency (*M*=5.29, *SD*=1.18) compared to the private embarrassment group (*M*=4.91, *SD*=1.34), *t*(166)=1.34, *p*=.183, and more compared to the control condition (*M*=4.62, *SD*=1.37), *t*(166)=2.83, *p*=.005. The private embarrassment group did not significantly differ from the control condition, *t*(166)=1.15, *p*=.245.
